# Prehospital use of point-of-care tests by community health workers: a scoping review

**DOI:** 10.3389/fpubh.2024.1360322

**Published:** 2024-04-24

**Authors:** Daniel Ebbs, Max Tarica, Melissa C. Funaro, Maggie O’Daniel, Michael Cappello

**Affiliations:** ^1^Department of Pediatrics, Yale University, New Haven, CT, United States; ^2^Department of Medicine, Harvey Cushing/John Hay Whitney Medical Library, New Haven, CT, United States; ^3^University of North Carolina at Greensboro, Greensboro, NC, United States; ^4^Department of Epidemiology of Microbial Diseases, Yale School of Public Health, New Haven, CT, United States

**Keywords:** community health worker, point-of-care test, mobile health, rapid diagnostic test, lay health worker

## Abstract

**Introduction:**

Point-of-Care Tests (POCTs) are utilized daily in resource abundant regions, however, are limited in the global south, particularly in the prehospital setting. Few studies exist on the use of non-malarial POCTs by Community Health Workers (CHWs). The purpose of this scoping review is to delineate the current diversity in and breadth of POCTs evaluated in the prehospital setting.

**Methods:**

A medical subject heading (MeSH) analysis of known key articles was done by an experienced medical librarian and scoping searches were performed in each database to capture “point of care testing” and “community health workers.” This review was guided by the PRISMA Extension for scoping reviews.

**Results:**

2735 publications were returned, 185 were nominated for full-text review, and 110 studies were confirmed to meet study criteria. Majority focused on malaria (74/110; 67%) or HIV (25/110; 23%); 9/110 (8%) described other tests administered. Results from this review demonstrate a broad geographic range with significant heterogeneity in terminology for local CHWs.

**Conclusion:**

The use of new POCTs is on the rise and may improve early risk stratification in limited resource settings. Current evidence from decades of malaria POCTs can guide future implementation strategies.

## 1 Introduction

The use of point-of-care tests (POCT) in the healthcare setting has exponentially advanced in the last decade given the number of novel biomarkers discovered with potential clinical significance (1–4). Advanced POCTs are utilized daily in resource abundant regions, however, are limited in the global south, particularly in the prehospital setting (1, 2, 5). Community health worker (CHW) programs often employ POCTs for rapid malaria diagnosis, however, there are few studies on the use of other non-malarial POCTs by CHWs (5–8). Additionally, although various training curricula and methods exist, no standardized evaluation process to assess POCT competency has been published.

CHWs are traditionally community members without formal training in healthcare who serve as appointed health representatives for frontline community health concerns, including emergencies (5, 9, 10). Heterogeneity between the definitions and roles of CHWs exists across the globe; CHWs are often trained to meet specific community health needs of a local population, and in low-and middle-income countries (LMIC) serve as the liaison between village and clinic (5, 9, 10). This is critical in LMICs, where it is common to have fewer than 1 physician per 100,000 residents (10, 11). Some programs have standardized the role of CHWs for subsets of patients; for example, the World Health Organization and United Nations Children’s Fund recommended the deployment of integrated community case management (iCCM) programs in 2004 that focus on prehospital evaluation and management of sick children (6–8). A component of the iCCM is the prehospital diagnosis of malaria using a rapid diagnostic test (RDT). Programs such as the iCCM bring training to CHWs and expand early access to emergency triage and treatment to extremely rural regions across the world (6–8). These regions typically have the highest pediatric and maternal mortality rates (6–8).

mHealth, a form of electronic health (eHealth) that uses portable technology such as cellular phones and tablet computers, is one method used to disseminate public health education in limited resource settings (12–15). Pedagogically, mHealth can combine multiple learning styles and apply innovative forms of education tailored to specific populations (14–17). Further, mHealth empowers its users and can harness the power of technology to provide culturally tailored and linguistically appropriate health education to rural communities (16, 17). Historically, mHealth has been underutilized due to the infrastructure required to purchase, connect, and maintain portable devices; however, over the last decade its cost has dropped significantly concurrent with the development of infrastructure for electricity, cellular service, and internet (14, 15). mHealth applications can provide more than innovative forms of education. Step-by-step algorithms for clinical decision management with POCTs have been developed with mHealth, applying current standard of care guidelines for various diseases (14–17). For example, an application can guide its users to input cardiovascular risk data points and serve as a screening tool for patients (16, 17). This can be expanded and tailored to community health workers in LMICs, where both chronic and neglected tropical diseases significantly impact healthy living (16, 17).

Similarly, medical technological advances that leapfrog from well-resourced industrial networks to low health resource settings hold the capacity to transform healthcare systems in LMICs (1–3, 16–18). For example, the development of portable ultrasound technology to identify high risk deliveries in rural villages exemplifies how technology can help decrease health disparity (18, 19). This allows a population’s socioeconomic status to improve by reducing unnecessary death and disability. POCTs are no different. A POCT is a test that can be completed at the patient’s bedside with nearly immediate results, all of which implies advanced portability (1, 2). With POCTs, the early diagnosis of serious illness improves triage decision-making and early treatment of infectious diseases (1–3). Malaria is a prime example. The clinical utility in detecting prehospital malaria with a rapid diagnostic test (RDT), a POCT, allows for prehospital providers to initiate life-saving treatment in their communities (6–8). Given the lack of health resources and potential impact of advancing POCTs in LMICs, this review will outline the current diversity in and breadth of POCTs that have been evaluated in the prehospital setting.

## 2 Methods

### 2.1 Search strategy

An experienced medical librarian (MCF) was consulted on methodology. A medical subject heading (MeSH) analysis [mesh.med.yale.edu] of known key articles was completed and scoping searches performed in each database. An iterative process was used to translate and refine searches. To maximize sensitivity, the formal search used controlled vocabulary terms and synonymous free-text words to capture the concepts of “point of care testing” and “community health workers.” The search strategy was peer reviewed by a second librarian, not otherwise associated with the project, using the PRESS standard (20). This scoping review is informed by the framework described by the Joanna Briggs Institute (JBI) (21). In addition, the reporting of this scoping review was guided by the PRISMA Extension for Scoping Reviews (PRISMA-ScR): Checklist and Explanation (22).

On June 21, 2022, the librarian performed a comprehensive search of multiple databases: MEDLINE, Embase, PsycInfo, Global Health, CINAHL, Web of Science, and Cochrane. To capture recently published articles, a second database search was repeated on September 13, 2023. No date or language limits were imposed on the search. All search strategies are included in Supplementary material. The final search retrieved a total of 2,735 results which were pooled in EndNote 20^1^ and de-duplicated using the Yale Reference Deduplicator [library.medicine.yale.edu/reference-deduplicator]. This set was uploaded to Covidence^2^ for screening.

### 2.2 Inclusion/exclusion criterion

Articles were included if POCTs were administered:

- in the prehospital/clinic setting
- by an individual in the same community

Participants may work both in a healthcare setting and as a community health worker; but must be providing POCT at the community level for inclusion. No age or gender was excluded. The overarching concept of interest for this scoping review is POCTs administered by community health workers in out of hospital environments for any medical purposes.

### 2.3 Selection of sources of evidence

Two screeners independently reviewed the titles, abstracts and full text of the eligible articles that met inclusion criteria. Any conflicts were resolved through consensus.

## 3 Results

**Figure 1:**
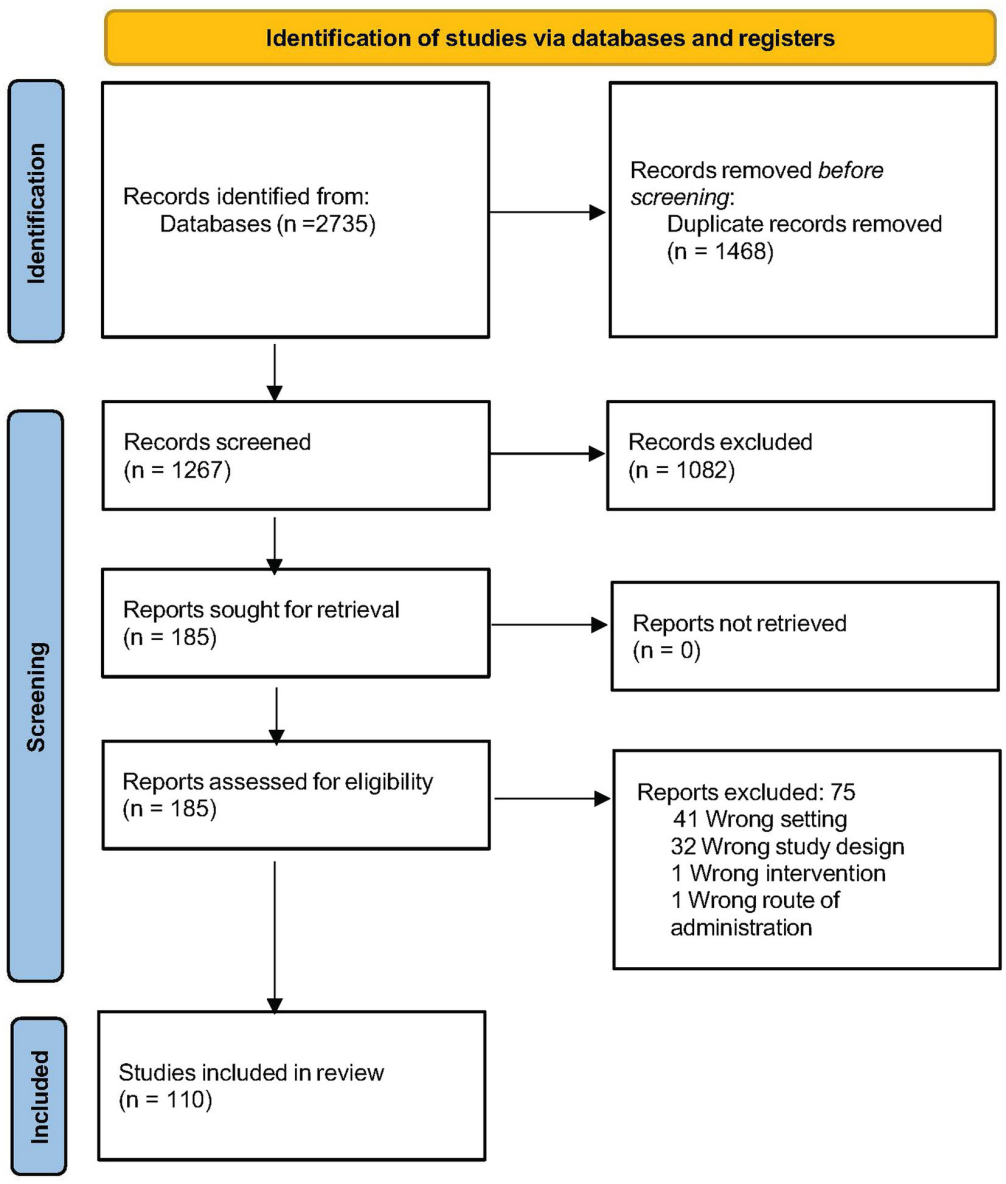
Review process.

Queries across multiple data bases returned 2,735 publications, of which 1,468 were duplicates. After a two-stage screening process, 185 potentially relevant articles were nominated for full-text review, after which 110 studies were confirmed to meet study criteria and underwent qualitative analysis (Flow Chart in Figure 1) (16, 24–133).

### 3.1 Heterogeneity in language used for the point-of-care test administrator

Among the included studies, there were various names used to refer to non-medical professionals trained to employ POCTs in the pre-hospital setting. Over 50% of included studies used the terminology “Community Health Worker,” with a smaller but substantial minority of studies using other terms, including Village Health Worker (VHW), Village Malaria Worker (VMW), Peers, Lay Health Provider, Lay Health Worker, and Community Health Volunteer.

### 3.2 Types of point-of-care tests employed

Ninety-nine of the 110 POCTs utilized in the included studies tested for malaria and HIV. Of the POCTs designated as “other,” these included the routine health markers blood glucose, hemoglobin A1c, cholesterol, and hemoglobin, as well as diagnostics for infectious pathogens such as Loa Loa, Schistosomes, SARS-COV-2 and Leishmania (37, 43, 47, 74, 83, 92, 107, 109). Additionally, one abstract evaluated the use of G6PD as a POCT (100). All 9 articles for ‘other’ POCTs were published in the last 10 years, with four in the last 3 years (Table 1). Three articles explicitly described skill retention for the POCT as followed over a set time-period.

**Table 1:** Nine non-malarial, other point of care tests (POCTs).

Year	Point of care test	Community health worker terminology	Type of study	Retention Eval.	Country
2018	HbA1c, Cholesterol, Creatinine	Community Healthcare Workers	Cross-Sectional	No	Haiti
2018	Hemoglobin	Community Health Worker (CHW)	Prospective	No	Pakistan
2015	Blood Glucose	Village Healthcare Worker	Prospective/Trial	No	India
2013	Cholesterol	Lay Community Volunteers	Prospective	No	Canada
2018	Schistosomiasis	Community Health Worker (CHW)	Clinical Trial	Yes	Tanzania
2021	Leishmania	Community Health Worker (CHW)	Cross-Sectional	No	Colombia
2022	SARS-CoV-2	Community Health Worker (CHW)	Cross-Sectional	No	Bangladesh
2020	Onchocerciasis	Community Health Worker (CHW)	Prospective	Yes	Cameroon
2020	G6PD	Village Malaria Workers	Prospective	Yes	Cambodia

#### 3.2.1 Routine health marker POCTs

Degennaro et al. evaluated the community diagnosis of non-communicable diseases, specifically the prevalence of chronic kidney disease, hyperlipidemia, and diabetes among communities in rural and urban Haiti (43). This is a unique, cross-sectional study that demonstrates prevalence of common non-communicable diseases and the successful application of community-based screening via CHWs. The article described a one-week training for the CHWs and limited details on the use of the POCTs which required a finger-stick blood sample.

McCormack et al. trained CHWs to obtain hemoglobin POCTs among a rural mountain community in Pakistan (47). CHWs diagnosed and treated community members who met the diagnostic criteria for anemia. After 8 weeks, repeat hemoglobin levels were obtained via POCTs to evaluate their intervention (education, mebendazole, oral iron). This study found a significant baseline prevalence of anemia among women (53%) and children (47%), as well as a significant reduction in anemia post-intervention.

Raghu et al. developed and piloted SMARTHealth, a mobile point-of-care clinical decision support tool in rural India, designed to facilitate the identification and management of cardiovascular disease in regions with limited health resources (107). This pilot study included the point-of-care measurement of blood glucose by Village Healthcare Workers via capillary puncture. This pilot was subsequently expanded to a stepped-wedge cluster randomized clinical trial that included blood glucose POCTs administered by Village Healthcare Workers.

Jones et al. evaluated cardiovascular risk factors in a high-risk group of South Asian Canadians in Calgary managed by lay community volunteers and applied community based participatory methodology (92). Lay community volunteers completed cardiovascular health assessments that included prehospital point-of-care cholesterol screening. Unique to this study is the screening sites, which were local religious facilities; additionally, this is the only non-HIV, non-malaria, ‘other’ prehospital POCT published in North America.

#### 3.2.2 Infectious disease POCT

Mazigo et al. introduced the first published prehospital POCT for diagnosing a trematode parasitic infection, Schistosomiasis, via *circulating cathodic antigen* (74). This cluster community randomized clinical trial proposes an alternate to mass drug administration for Schistosomiasis: a diagnostic test first and treat if recommended approach. This is a clinical trial protocol and data from this clinical trial is yet to be published.

Cossio et al. completed the first study evaluating the early, ‘community’ diagnosis of cutaneous leishmaniasis in Colombia via a method they developed, *Isothermal Recombinase Polymerase Amplification* targeting *Leishmania* kinetoplast DNA combined with lateral flow immunochromatography (109). A reference laboratory reviewed all samples and were blinded to prehospital results. Study findings demonstrated similar accuracy in diagnosis and highlighted the feasibility of this test to be conducted by CHWs in a community setting.

Sania et al. trained CHWs to complete SARS-COV-2 rapid antigen testing via nasal/saliva swabs in low-income communities near Dhaka, Bangladesh (37). This timely cross-sectional study addressed barriers to access to care by bringing testing directly to communities and their families via CHWs. The authors noted that by introducing CHWs for POCT administration, testing of symptomatic patients with concern for SARS-COV-2 increased fourfold. Notably, nasal swabs demonstrated higher accuracy than saliva samples.

Pion et al. published the first prehospital POCT to evaluate parasite load (concentration of microfilariae) prior to onchocerciasis treatment (83). Given that adverse events typically occur with high levels of microfilaremia (>20,000 per ml), the authors described the use of a novel tool to detect prehospital concentrations prior to treatment: the *LoaScope.* This study concluded that for individuals with previous microfilariae concentrations <20,000 per ml, and treated with ivermectin, retesting is not required given minimal risk of substantial change over 2 years. Therefore, if patients are tracked, treatment protocols in the future can be streamlined without testing in the community via CHWs.

#### 3.2.3 G6PD POCT

Patients with glucose-6-phosphate dehydrogenase deficiency (G6PD) are at risk for serious hemolysis when treated for *Plasmodium vivax* with primaquine. Given this risk, Wojnarski et al. deployed a G6PD POCT via Village Malaria Workers in rural Cambodia (100). This study is the first published that utilizes a prehospital G6PD POCT and demonstrated reliable accuracy in results among prehospital vs. laboratory settings.

### 3.3 Geographical distribution of prehospital point-of-care testing

**Figure 2:**
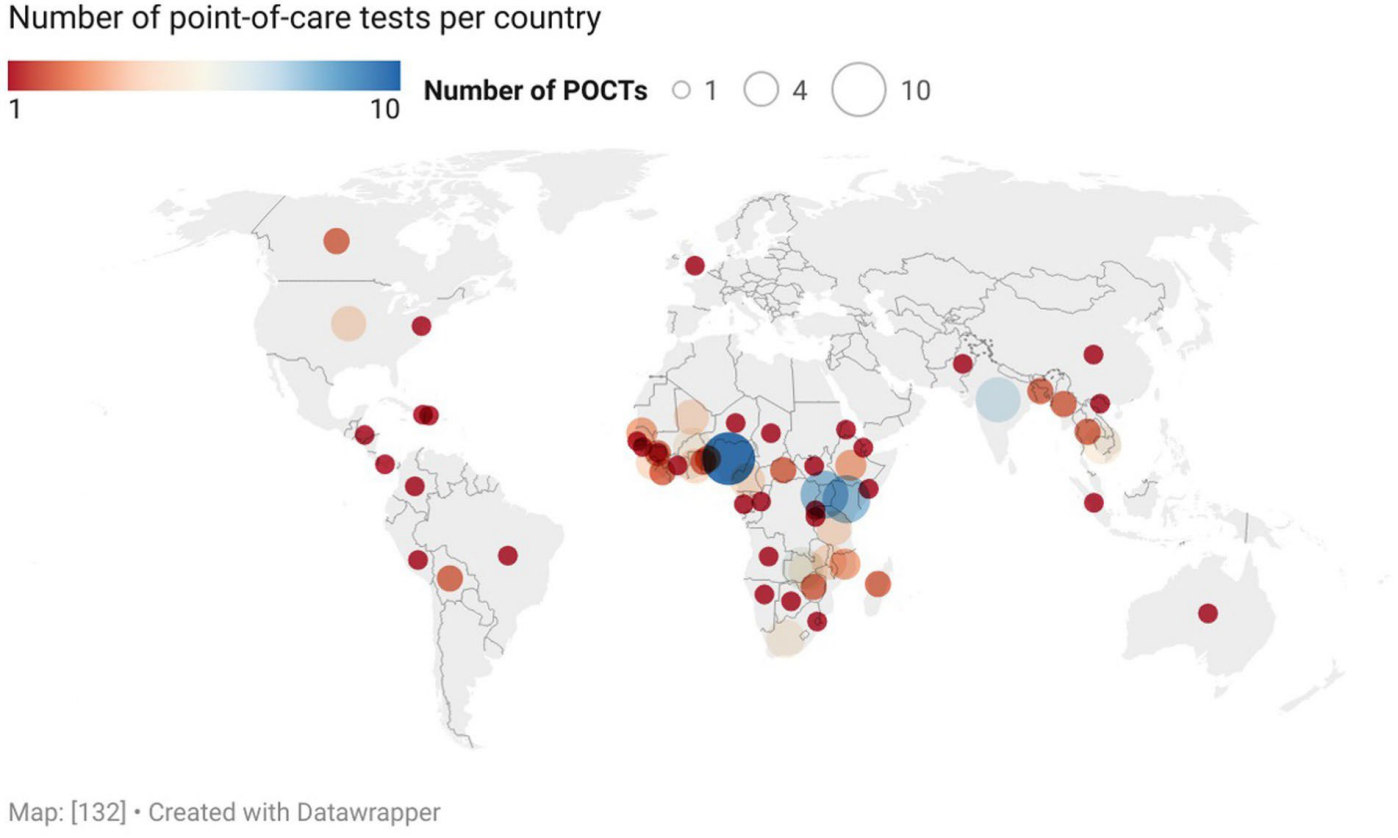
Prehospital POCTs per country.

Review of country-level locations of study interventions showed that a majority of POCT evaluations occurred in the Global South (Figure 2) (134). Specifically, in decreasing order by continent, 67 studies occurred in Africa (predominantly in the Sub-Saharan region), 24 in Asia (predominantly in Southeast Asia), 10 in North America (including Caribbean islands and Central American countries), 5 in South America, 1 in Europe, 1 in Australia, and 2 in unspecified areas.

### 3.4 Type of study design

Articles were characterized by type; for prehospital POCTs, 56/110 (51%) of articles were prospective studies without clinical trials, 10/110 (9%) were clinical trials, and 29/110 (26%) were abstracts. Of note, we reviewed and included three clinical trial protocols that met inclusion criterion. None of the 29 abstracts were non-malaria, non-HIV POCTs (Table 1).

## 4 Discussion

Simultaneous to the rapid development of novel biomarkers for disease, the capacity to implement POCTs at the community level has changed (1, 6, 7). The findings in this review demonstrate that a diversity of non-malarial prehospital POCTs are being utilized to identify endemic diseases in specific geographic regions. The review also determined that prehospital POCTs are primarily being studied in LMICs. Finally, this review outlines the need for a standardized approach to implementation, monitoring, and evaluation of new POCTs and finds significant heterogeneity between prehospital provider terminology globally.

Although only 11 studies in this review were focused on non-malaria, non-HIV POCTs, 10 (91%) of these studies were published in the last 10 years, and eight (73%) of these in the last 5 years. Portability, decreased cost, and wider accessibility aligned with ease of use in the prehospital setting, has promoted the use of POCTs outside the hospital setting. However, the clinical utility of prehospital POCTs remains unknown. Practical considerations and expert opinion both advocate in favor of implementing POCTs in LMICs; however, after decades of malaria RDT use and subsequent treatment in the prehospital setting, many challenges were identified (1, 6–8). Particularly, financial constraints, limits to supervision and monitoring of CHWs, and a paucity of evidence related to clinical outcomes, constrains progress and possible transformation adaptation (5–8, 23). To improve evaluation for navigating these complexities, a recent content analysis of iCCM programs proposed several frameworks to manage and evaluate programs (23). This overview allows program managers to prepare for process evaluation, sustainability, adequate monitoring, and outcome measurements. This is the necessary first-step in overcoming the many barriers to outcomes research for prehospital POCTs in LMICs.

Primary care in limited health resource settings can be minimal to nonexistent (135). With aging populations, chronic disease becomes more prevalent and there is a need for innovative solutions to address this need in LMICs (135, 136). mHealth offers a unique and timely solution. mHealth applications that use stepwise clinical decision algorithms can incorporate risk factors and POCTs (136). Three studies in this review evaluated risk factors for cardiovascular disease with the input of POCTs (43, 92, 107). Raghu et al. trialed a SMARTHealth mobile POCT application that uses a clinical decision support tool to help guide prehospital providers (107). With advances in telecommunication, decision tools of the alike can be tailored to communities with real-time referral of patients who are at risk and require medical attention. Additionally, telehealth can be integrated into this framework; clinicians may review records and provide online consultation for diagnosis, education, and treatment at the community level. Both Jones et al. and Degennaro et al. also integrated prehospital POCTs to evaluate risk factors and potential benefit of prehospital screening, albeit without a mHealth component (43, 92).

**Figure 3:**
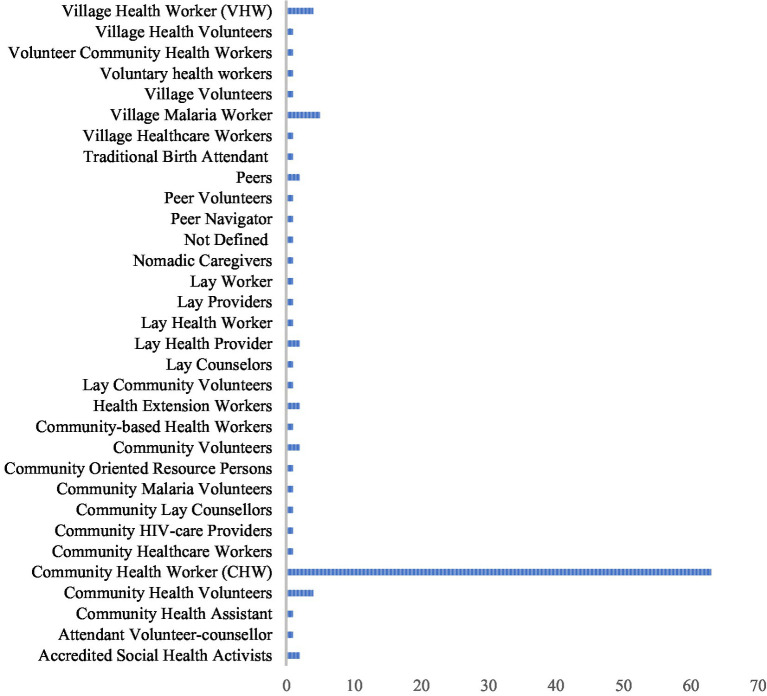
Prehospital terminology.

Under the umbrella definition used to identify a CHW in this review, over 30 alternate community definitions were discovered (Figure 3). The majority of CHWs are appointed by local leaders or a quorum of community members to serve as health access points among their communities; the heterogeneity in specific terminology has deep cultural and historical context (8, 9). This variability in terminology is critical to note for future studies or reviews, as standardization is imperative when evaluating prehospital providers in different regions of the world. Further investigation on this variability can help identify potential influences on POCT successes or failures.

This review is limited to available manuscripts and may underrepresent the current diversity in prehospital POCTs. CHW training programs exist across the world and may not have the resources or current interest in publishing on POCT use. This is more limiting for standard POCTs like malaria RDTs, as novel POCTs generally can be identified in early implementation stages when their utility is under investigation. This review included trial protocols and abstracts to capture POCTs that may not be widely disseminated and/or are in development.

This is the first scoping review to evaluate the use of prehospital POCTs by CHWs. The findings in this review demonstrate that prehospital POCTs have increased in number and type over the last two decades with a particular focus on novel, ‘other’ POCTs evaluated over the last 5 years. This review also highlights the under-utilization of mHealth in LMICs and the potential impact of clinical decision support tools that incorporate POCTs for chronic disease management. Overall, it is imperative that POCT use is standardized in the prehospital setting in LMICs, as this may bring early access to care to marginalized communities.

## Author contributions

DE: Conceptualization, Data curation, Formal analysis, Investigation, Methodology, Project administration, Resources, Software, Supervision, Validation, Writing – original draft, Writing – review & editing. MT: Investigation, Writing – original draft. MF: Data curation, Formal analysis, Resources, Software, Writing – original draft. MO’D: Writing – original draft. MC: Resources, Supervision, Writing – review & editing.

## Footnotes

^1^ www.endnote.com

^2^ www.covidence.org
