# Correction: Prehospital use of point-of-care tests by community health workers: a scoping review

**DOI:** 10.3389/fpubh.2026.1799389

**Published:** 2026-02-20

**Authors:** Daniel Ebbs, Max Tarica, Melissa C. Funaro, Maggie O'Daniel, Michael Cappello

**Affiliations:** 1 Department of Pediatrics, Yale University, New Haven, CT, United States; 2 Department of Medicine, Harvey Cushing/John Hay Whitney Medical Library, New Haven, CT, United States; 3 University of North Carolina at Greensboro, Greensboro, NC, United States; 4 Department of Epidemiology of Microbial Diseases, Yale School of Public Health, New Haven, CT, United States

**Keywords:** community health worker, point-of-care test, mobile health, rapid diagnostic test, lay health worker

In the published article, an author name was incorrectly written as Max Taricia. The correct spelling is Max Tarica.

The original article has been updated.

