# Supplementary material for: Prehospital use of point-of-care tests by community health workers: a scoping review
Source: Front Public Health. 2024 Apr 24;12:1360322. doi: 10.3389/fpubh.2024.1360322 (PMC11076783; doi:10.3389/fpubh.2024.1360322)
Supplement: Supplementary file 1 [file Data_Sheet_1.docx]

Supplementary Material

Prehospital Use of Point-of-Care Tests by Community Health Workers: a Scoping Review

Ebbs, Daniel*, Taricia, Max, Funaro, Melissa, O’Daniel, Maggie, Cappello, Michael

*** Correspondence:** Daniel.ebbs@yale.edu

# Supplementary Data

Supplement 1: Search strategies for all databases

Ovid MEDLINE(R) ALL

| 1 | "Point of Care Systems"/ or "Point of Care Testing"/ or (("point of care" or "point of care" or poc or rapid or bedside) adj5 (device* or diagnos* or laborator* or technolog* or comput* or analyzer or analyser or test* or analys* or immunoassay* or technique* or immunofluorescence or "fluorescent antibody")).tw,kw. |
| --- | --- |
| 2 | Community Health Aides/ or Home Health Aides/ or Allied Health Personnel/ or Voluntary Workers/ or Home Nursing/ or Peer Group/ or Social Support/ |
| 3 | ((lay or voluntary or volunteer* or untrained or unlicensed or nonprofessional* or non professional*) adj5 (worker* or visitor* or attendant* or aide or aides or support* or person* or helper* or carer* or caregiver* or care giver* or consultant* or assistant* or staff or visit* or midwife or midwives)).tw,kw. |
| 4 | ((Lay or peer) adj volunteer*).tw,kw. |
| 5 | (trained adj3 (volunteer* or health worker*)).tw,kw. |
| 6 | ((community or village*) adj3 (health worker* or health care worker* or healthcare worker*)).tw,kw. |
| 7 | (community adj3 (volunteer* or aide or aides or support)).tw,kw. |
| 8 | (paraprofessional? or paramedic or paramedics or paramedical worker? or paramedical personnel or allied health personnel or allied health worker? or support worker? or home health aide?).tw,kw. |
| 9 | or/2-8 |
| 10 | 1 and 9 |

Embase (Ovid)

| 1 | point of care testing/ or (("point of care" or "point of care" or poc or rapid or bedside) adj5 (device* or diagnos* or laborator* or technolog* or comput* or analyzer or analyser or test* or analys* or immunoassay* or technique* or immunofluorescence or "fluorescent antibody")).tw,kw. |
| --- | --- |
| 2 | Voluntary Worker/ or Health Auxiliary/ or Peer Group/ or Health Visitor/ |
| 3 | ((lay or voluntary or volunteer* or untrained or unlicensed or nonprofessional* or non professional*) adj5 (worker* or visitor* or attendant* or aide or aides or support* or person* or helper* or carer* or caregiver* or care giver* or consultant* or assistant* or staff or visit* or midwife or midwives)).tw,kw. |
| 4 | ((Lay or peer) adj volunteer*).tw,kw. |
| 5 | (trained adj3 (volunteer* or health worker*)).tw,kw. |
| 6 | ((community or village*) adj3 (health worker* or health care worker* or healthcare worker*)).tw,kw. |
| 7 | (community adj3 (volunteer* or aide or aides or support)).tw,kw. |
| 8 | (paraprofessional? or paramedic or paramedics or paramedical worker? or paramedical personnel or allied health personnel or allied health worker? or support worker? or home health aide?).tw,kw. |
| 9 | or/2-8 |
| 10 | 1 and 9 |
| 11 | limit 10 to conference abstracts |
| 12 | 10 not 11 |

Global Health (Ovid)

| 1 | (("point of care" or "point of care" or poc or rapid or bedside) adj5 (device* or diagnos* or laborator* or technolog* or comput* or analyzer or analyser or test* or analys* or immunoassay* or technique* or immunofluorescence or "fluorescent antibody")).tw. |
| --- | --- |
| 2 | exp community health workers/ or home health aides/ |
| 3 | ((lay or voluntary or volunteer* or untrained or unlicensed or nonprofessional* or non professional*) adj5 (worker* or visitor* or attendant* or aide or aides or support* or person* or helper* or carer* or caregiver* or care giver* or consultant* or assistant* or staff or visit* or midwife or midwives)).tw. |
| 4 | ((Lay or peer) adj volunteer*).tw. |
| 5 | (trained adj3 (volunteer* or health worker*)).tw. |
| 6 | ((community or village*) adj3 (health worker* or health care worker* or healthcare worker*)).tw. |
| 7 | (community adj3 (volunteer* or aide or aides or support)).tw. |
| 8 | (paraprofessional? or paramedic or paramedics or paramedical worker? or paramedical personnel or allied health personnel or allied health worker? or support worker? or home health aide?).tw. |
| 9 | or/2-8 |
| 10 | 1 and 9 |

CINAHL Complete (EBSCOhost)

| S12 | S3 AND S11 |
| --- | --- |
| S11 | S4 OR S5 OR S6 OR S7 OR S8 OR S9 OR S10 |
| S10 | TI ( paraprofessional* or paramedic or paramedics or paramedical or "allied health personnel" or "allied health worker*" or "support worker*" or "home health aide*") OR AB ( paraprofessional* or paramedic or paramedics or paramedical or "allied health personnel" or "allied health worker*" or "support worker*" or "home health aide*" ) |
| S9 | TI ( (community N3 (volunteer* or aide or aides or support)) ) OR AB ( (community N3 (volunteer* or aide or aides or support)) ) |
| S8 | TI ( ((community or village*) N3 (health worker* or health care worker* or healthcare worker*)) ) OR AB ( ((community or village*) N3 (health worker* or health care worker* or healthcare worker*)) ) |
| S7 | TI ( (trained N3 (volunteer* or health worker*)) ) OR AB ( (trained N3 (volunteer* or health worker*)) ) |
| S6 | TI ( (Lay or peer) N volunteer* ) OR AB ( (Lay or peer) N volunteer* ) |
| S5 | TI ( (  (lay or voluntary or volunteer* or untrained or unlicensed or nonprofessional* or non professional*) N5 (worker* or visitor* or attendant* or aide or aides or support* or person* or helper* or carer* or caregiver* or care giver* or consultant* or assistant* or staff or visit* or midwife or midwives)) ) OR AB ( ((lay or voluntary or volunteer* or untrained or unlicensed or nonprofessional* or non professional*) N5 (worker* or visitor* or attendant* or aide or aides or support* or person* or helper* or carer* or caregiver* or care giver* or consultant* or assistant* or staff or visit* or midwife or midwives)) ) |
| S4 | MH Volunteer Workers OR MH Health Personnel, Unlicensed OR MH Home Health Aides OR MH Allied Health Personnel OR MH Community Health Workers OR MH Nursing Assistants OR MH Caregivers OR MH Peer Group OR MH Community Networks |
| S3 | S1 OR S2 |
| S2 | TI ( (("point of care" or "point of care" or poc or rapid or bedside) N5 (device* or diagnos* or laborator* or technolog* or comput* or analyzer or analyser or test* or analys* or immunoassay* or technique* or immunofluorescence or "fluorescent antibody")) ) OR AB ( (("point of care" or "point of care" or poc or rapid or bedside) N5 (device* or diagnos* or laborator* or technolog* or comput* or analyzer or analyser or test* or analys* or immunoassay* or technique* or immunofluorescence or "fluorescent antibody")) ) |
| S1 | (MH "Point‐of‐Care Testing") |

Web of Science Core Collection (Clarivate)

| 9 | #8 AND #1 |
| --- | --- |
| 8 | #7 OR #6 OR #5 OR #4 OR #3 OR #2 |
| 7 | TI=(paraprofessional* or paramedic or paramedics or paramedical or "allied health personnel" or "allied health worker*" or "support worker*" or "home health aide*") OR AB=(paraprofessional* or paramedic or paramedics or paramedical or "allied health personnel" or "allied health worker*" or "support worker*" or "home health aide*") OR AK=(paraprofessional* or paramedic or paramedics or paramedical or "allied health personnel" or "allied health worker*" or "support worker*" or "home health aide*") |
| 6 | TI=(community NEAR/3 (volunteer* or aide or aides or support)) OR AB=(community NEAR/3 (volunteer* or aide or aides or support)) OR AK=(community NEAR/3 (volunteer* or aide or aides or support)) |
| 5 | TI=((community or village*) NEAR/3 ("health worker*" or "health care worker*" or "healthcare worker*")) OR AB=((community or village*) NEAR/3 ("health worker*" or "health care worker*" or "healthcare worker*")) OR AK=((community or village*) NEAR/3 ("health worker*" or "health care worker*" or "healthcare worker*")) |
| 4 | TI=(trained NEAR/3 (volunteer* or "health worker*")) OR AB=(trained NEAR/3 (volunteer* or "health worker*")) OR AK=(trained NEAR/3 (volunteer* or "health worker*")) |
| 3 | TI=((Lay or peer) NEAR volunteer* ) OR AB=((Lay or peer) NEAR volunteer* ) OR AK=((Lay or peer) NEAR volunteer* ) |
| 2 | TI=((lay or voluntary or volunteer* or untrained or unlicensed or nonprofessional* or "non professional*") NEAR/5 (worker* or visitor* or attendant* or aide or aides or support* or person* or helper* or carer* or caregiver* or "care giver*" or consultant* or assistant* or staff or visit* or midwife or midwives)) OR AB=((lay or voluntary or volunteer* or untrained or unlicensed or nonprofessional* or "non professional*") NEAR/5 (worker* or visitor* or attendant* or aide or aides or support* or person* or helper* or carer* or caregiver* or "care giver*" or consultant* or assistant* or staff or visit* or midwife or midwives)) OR AK=((lay or voluntary or volunteer* or untrained or unlicensed or nonprofessional* or "non professional*") NEAR/5 (worker* or visitor* or attendant* or aide or aides or support* or person* or helper* or carer* or caregiver* or "care giver*" or consultant* or assistant* or staff or visit* or midwife or midwives)) |
| 1 | TI=((("point of care" or "point of care" or poc or rapid or bedside) NEAR/5 (device* or diagnos* or laborator* or technolog* or comput* or analyzer or analyser or test* or analys* or immunoassay* or technique* or immunofluorescence or "fluorescent antibody"))) OR AB=((("point of care" or "point of care" or poc or rapid or bedside) NEAR/5 (device* or diagnos* or laborator* or technolog* or comput* or analyzer or analyser or test* or analys* or immunoassay* or technique* or immunofluorescence or "fluorescent antibody"))) OR AK=((("point of care" or "point of care" or poc or rapid or bedside) NEAR/5 (device* or diagnos* or laborator* or technolog* or comput* or analyzer or analyser or test* or analys* or immunoassay* or technique* or immunofluorescence or "fluorescent antibody"))) |

Cochrane (Wiley)

| #1 | (("point of care" or "point of care" or poc or rapid or bedside) NEAR/5 (device* or diagnos* or laborator* or technolog* or comput* or analyzer or analyser or test* or analys* or immunoassay* or technique* or immunofluorescence or "fluorescent antibody")):ti,ab,kw |
| --- | --- |
| #2 | ((lay or voluntary or volunteer* or untrained or unlicensed or nonprofessional* or non NEXT professional*) NEAR/5 (worker* or visitor* or attendant* or aide or aides or support* or person* or helper* or carer* or caregiver* or care NEXT giver* or consultant* or assistant* or staff or visit* or midwife or midwives)):ti,ab,kw |
| #3 | ((Lay or peer) NEAR volunteer*):ti,ab,kw |
| #4 | (trained NEAR/3 (volunteer* or health NEXT worker*)):ti,ab,kw |
| #5 | ((community or village*) NEAR/3 (health NEXT worker* or "health care worker*" or "healthcare worker*")):ti,ab,kw |
| #6 | (community NEAR/3 (volunteer* or aide or aides or support)):ti,ab,kw |
| #7 | (paraprofessional* or paramedic or paramedics or paramedical or "allied health personnel" or "allied health worker" or "allied health workers" or support NEXT worker* or "home health aide" or "home health aides"):ti,ab,kw |
| #8 | #7 OR #6 OR #5 OR #4 OR #3 OR #2 |
| #9 | #8 AND #1 |

**
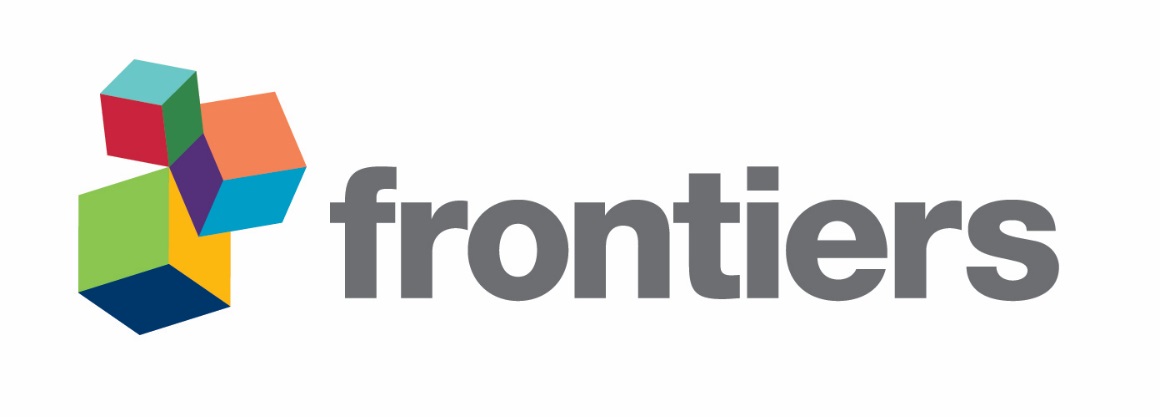
**
